# “*It gives me the strength and courage to take care of myself”*: a qualitative exploration of experiences with STI testing among women who initiated PrEP during pregnancy in Western Kenya

**DOI:** 10.1002/jia2.26464

**Published:** 2025-04-23

**Authors:** Jerusha N. Mogaka, Tessa Concepcion, Felix Abuna, Eunita Akim, Chelsea Morroni, Aamirah Mussa, Melissa Mugambi, Helen Aketch, Sarah Obatsa, Allison R. Webel, John Kinuthia, Kenneth Ngure, Kristin M. Beima‐Sofie, Grace John‐Stewart, Jillian Pintye

**Affiliations:** ^1^ School of Nursing University of Washington Seattle Washington USA; ^2^ Department of Global Health University of Washington Seattle Washington USA; ^3^ Research and Programs Department Kenyatta National Hospital Nairobi Kenya; ^4^ Botswana Harvard Health Partnership Gaborone Botswana; ^5^ MRC Centre for Reproductive Health The University of Edinburgh Edinburgh UK; ^6^ Department of Community Health Jomo Kenyatta University of Agriculture and Technology Nairobi Kenya

**Keywords:** PrEP, user experiences, sexually transmitted infections, STI testing, risk perception, pregnant and postpatum women

## Abstract

**Introduction:**

Sexually transmitted infections (STIs) in pregnancy contribute to poor perinatal outcomes and increased HIV acquisition risk, underscoring the importance of delivering STI/HIV services within antenatal care. Few studies evaluate women's perspectives on the co‐delivery of antenatal STI testing and HIV pre‐exposure prophylaxis (PrEP). We sought to understand motivations for and experiences with STI testing among pregnant women who initiated HIV PrEP.

**Methods:**

We conducted semi‐structured in‐depth interviews (IDIs) among a subset of women enrolled in a randomized trial in Western Kenya (NCT04472884) who initiated PrEP within antenatal clinics and tested for *Chlamydia trachomatis* (CT) and *Neisseria gonorrhoeae* (NG) in pregnancy and/or postpartum. As part of parent study procedures, IDIs were conducted between September 2023 and April 2024. Interviews were recorded, transcribed and thematically analysed using deductive and inductive methods. The Health Belief Model guided exploration of STI testing experiences, motivations for testing and the impact of testing on PrEP use.

**Results:**

Overall, 39 women who initiated PrEP during pregnancy and tested for CT/NG participated in IDIs; six tested positive for CT and/or NG. Median age was 26 years (IQR 21–29), 77% of participants had >8 years of education, 15% were employed and 72% were married. Most (86%) did not know their partner's HIV status, and 82% persisted with PrEP use at 9 months postpartum. Perceived vulnerability to STI/HIV acquisition, fear of adverse outcomes from untreated infections (e.g. pregnancy loss or harm to baby) and desire to alleviate symptoms (e.g. abnormal discharge) motivated STI testing uptake when offered during antenatal visits. Provision of STI‐related education, availability of STI services (i.e. immediate treatment, expedited partner therapy) and supportive interactions with providers promoted positive experiences with STI testing. STI testing encouraged health‐promoting behaviours, including sustained PrEP use, even when STI results were negative, as testing made women feel proactively involved in preventing HIV/STI complications for themselves and their infants.

**Conclusions:**

In this qualitative evaluation among women who initiated PrEP in pregnancy, STI testing encouraged PrEP use, even when results were negative. Incorporating STI testing within PrEP delivery in antenatal care represents an opportunity for addressing HIV/STI in this priority population.

## 1 INTRODUCTION

Pregnant women in sub‐Saharan Africa experience disproportionately high rates of curable sexually transmitted infections (STIs), with the prevalence of *Chlamydia trachomatis* (CT) and *Neisseria gonorrhoeae* (NG) reaching as high as 36.8% and 7.6%, respectively [1, 2, 3, 4]. If left untreated, these infections contribute to poor pregnancy and birth outcomes in these settings [5, 6]. Additionally, STIs increase the risk of HIV acquisition during pregnancy among women without HIV and the risk of vertical HIV transmission among women who acquire HIV during pregnancy [7, 8]. Therefore, simultaneously addressing HIV and other STIs is an important opportunity for improving global maternal and infant health. Pre‐exposure prophylaxis (PrEP) does not protect against curable STIs commonly detected among pregnant PrEP users, such as CT and NG [9, 10], and studies to address CT and NG in this population are few. Syndromic STI management is the standard of care in Kenya, resulting in delayed or missed treatment opportunities [11]. Integrating empiric STI testing within antenatal clinics offering PrEP services could address STIs and promote HIV prevention as a potentially high‐yield strategy for women at high risk for both. Pregnant women in Kenya generally underestimate their HIV risk [12]. Testing for STIs and/or a diagnosis of an STI may increase one's risk perception and cue preventive measures, including HIV PrEP. STI testing presents an opportunity to engage in discussions with health providers about HIV prevention strategies, making co‐delivery of STI testing with PrEP particularly attractive. Additionally, incorporating other STI screening into the current “opt‐out” model for HIV testing within antenatal care clinics may normalize testing and reduce stigma.

Several quantitative pilot studies among pregnant women living with and without HIV in South Africa, Botswana and Kenya demonstrate consistently high feasibility and acceptability (85−99%) of “near‐patient” Xpert® testing for CT, NG and *Trichomonas vaginalis* within routine antenatal settings [13, 14, 15]. Yet, few qualitative data exist from end‐users on STI testing service delivery integrated within antenatal care settings. Prior to the PrEP era, existing studies among general populations of pregnant women explored barriers and facilitators of STI testing and expedited partner therapy (EPT). These studies identified testing characteristics (e.g. ease of use, results turnaround time, integration of service delivery and EPT availability), client factors (e.g. STI knowledge, attitude and beliefs, relationship dynamics, pregnancy status, risk perception and self‐efficacy) and structural factors (e.g. access to services and resources and availability of guidelines) as important for implementation [13, 14, 16–20]. However, no studies to date have evaluated STI testing experiences among pregnant PrEP users who may have unique perspectives on STI testing, shaped by their behavioural profiles such as the need to protect themselves and infants as well as adoption of HIV preventive strategies such as PrEP [21]. Understanding experiences with STI testing among pregnant women who initiate PrEP is helpful to refine co‐delivery strategies in this unique population [22].

We conducted a prospective study exploring the burden of CT and/or NG among women who initiated oral PrEP during pregnancy in Western Kenya [23]. We found a prevalence of CT and/or NG at enrolment in pregnancy of 8.6% and a high incidence (11 infections per 100 person‐years) through 9 months postpartum, highlighting the burden of CT/NG among women who initiate PrEP [23]. We conducted this qualitative evaluation to understand women's experiences with CT/NG testing within antenatal clinics delivering PrEP and the impact of CT/NG testing on PrEP continuation among women who initiated PrEP during pregnancy. Our overall objective was to gather experiences and perspectives to inform co‐delivery of CT/NG testing within antenatal care settings offering PrEP for future implementation and scale‐up.

## 2 METHODS

### 2.1 Study design and population

We conducted a qualitative evaluation informed by the Health Belief Model [24]. We invited a subset of women enrolled in the ongoing mWACh‐PrEP study (NCT04472884) who were offered CT and NG testing using GeneXpert® CT/NG assays as part of parent study procedures to participate in in‐depth interviews (IDIs). The parent study, including STI testing activities, is detailed elsewhere [25, 26]. Briefly, the mWACh‐PrEP study is a randomized trial that tests a mHealth tool to improve PrEP adherence among pregnant women not living with HIV who newly initiate oral PrEP at five routine antenatal clinics in Siaya and Kisumu, Kenya. The prevalence of HIV in the region ranges from 14% to 16% [27] and the prevalence of other STIs like CT/NG is 5–10% [23, 28]. At a subset of two sites, we offered CT/NG testing at enrolment or at any point during pregnancy and at 6 and 9 months postpartum. Women enrolled in the trial before STI testing began were offered CT and NG testing at their next study visit. Women were instructed on self‐collection of vaginal swabs, with clinician‐assisted sample collection available for those uncomfore. Women testing positive for CT or NG were offered treatment at no cost. mWACh‐PrEP study participants were eligible for the current qualitative study if they were offered and accepted CT/NG testing at least once at any point during the parent study, either during pregnancy or postpartum. All women offered CT and NG testing accepted [25]. Oral PrEP use status was dichotomized (continued or discontinued) based on self‐report at 9 months postpartum.

### 2.2 Data collection

#### 2.2.1 Participant recruitment

Nurses affiliated with the mWACh‐PrEP study but not associated with or offering STI testing services informed participants about the qualitative sub‐study during their follow‐up visits. A subset of women was purposively selected after completion of follow‐up procedures at 9 months postpartum. We sought a heterogeneous group of women, including those who tested for CT and NG during pregnancy, postpartum or both time points, and those diagnosed with either CT and/or NG, to represent varied experiences with the STI services offered. Qualitative interviewers invited participants to join IDIs from September 2023 to April 2024.

#### 2.2.2 Data collection

Based on published literature and previous experience working with this population and region, JNM, TC, HA, CO and SO collaboratively created semi‐structured interview guides. EA, HA, CO and SO translated the interview guides into Swahili and Dholuo and then back‐translated them into English for quality control and to maintain the intended meaning.

Based on domains of the Health Belief Model, interview guides were developed to capture user experiences and motivations for STI testing, including individual factors (perceived threats and benefits), modifying factors (*psychosocial*), self‐efficacy (*competence*) and cues to actions (*triggers for change*). EA, TC and trained qualitative interviewers (HA, CO and SO) working within the region and familiar with the local context piloted the interview guides to ensure question comprehension and flow. Between September 2023 and April 2024, qualitative interviewers conducted audio‐recorded interviews in a private room within the study clinic, using the participant's preferred language (English, Kiswahili or Dholuo), with each session taking approximately 40 minutes. HA, CO and SO conducted data cleaning, verbatim transcription and translation into English when necessary.

An initial set of transcripts was independently verified against the audio recording by EA and JN, both fluent in the local languages. The qualitative team evaluated all transcripts for completeness and accuracy, while a random sample of 7 (18%) transcripts were back‐translated into Swahili and Dholuo for accuracy as part of the quality assurance and quality checks.

### 2.3 Data analysis

A codebook was developed by randomly selecting 10 transcripts and using both deductive and inductive thematic analysis approaches to test and iteratively generate potential codes related to CT and NG testing motivators and user experiences [29, 30]. We generated codes deductively based on the topic area guides and research questions, while inductive codes emerged from the transcript data. The coding process began with open coding and discussion within the core team, leading to a list of potential codes refined with subsequent discussions and review of additional transcripts iteratively. Consensus coding of a similar transcript was conducted by the whole coding team, followed by further discussions to refine the finalized codebook. Dedoose (https://www.dedoose.com/) was used for data management and analysis. Primary coding of the IDIs was conducted by JN, TC, EA, HA and SO. Each transcript was coded independently using the final revised codebook to ensure the rigour and reliability of findings per the consolidated criteria for reporting qualitative research (COREQ) guidelines. Memos facilitated discussions when reviewing divergent codes [31].

Secondary coding, which entailed a review of each other's codes to identify any disagreements, followed by discussions to resolve any divergent codes, was guided by memos developed during the coding process. After completion of the coding process, we generated initial themes by running queries on individual and overlapping codes to extract data and formulate a conceptual map that was used to connect related concepts within the Health Belief Model. The map was used to identify themes based on conceptual relationships. Data analysis was informed by the Health Belief Model, categorizing the constructs into modifying factors (knowledge and attitude towards STI testing), individual factors (perceived susceptibility, benefits, barriers and self‐efficacy) and cues to action that influence uptake of STI testing and PrEP use. The domains were then organized into the main topic areas of (1) individual motivations for CT and NG testing; (2) impact of CT and NG testing on PrEP use; and (3) recommendations for improving STI testing and treatment in antenatal care [24].

### 2.4 Ethical considerations

Study activities were approved by the Kenyatta National Hospital‐University of Nairobi Ethics and Research Committee (P319/05/2021) and the University of Washington Institutional Review Board (STUDY00010797).

## 3 RESULTS

### 3.1 Participant characteristics

**Figure 1:**
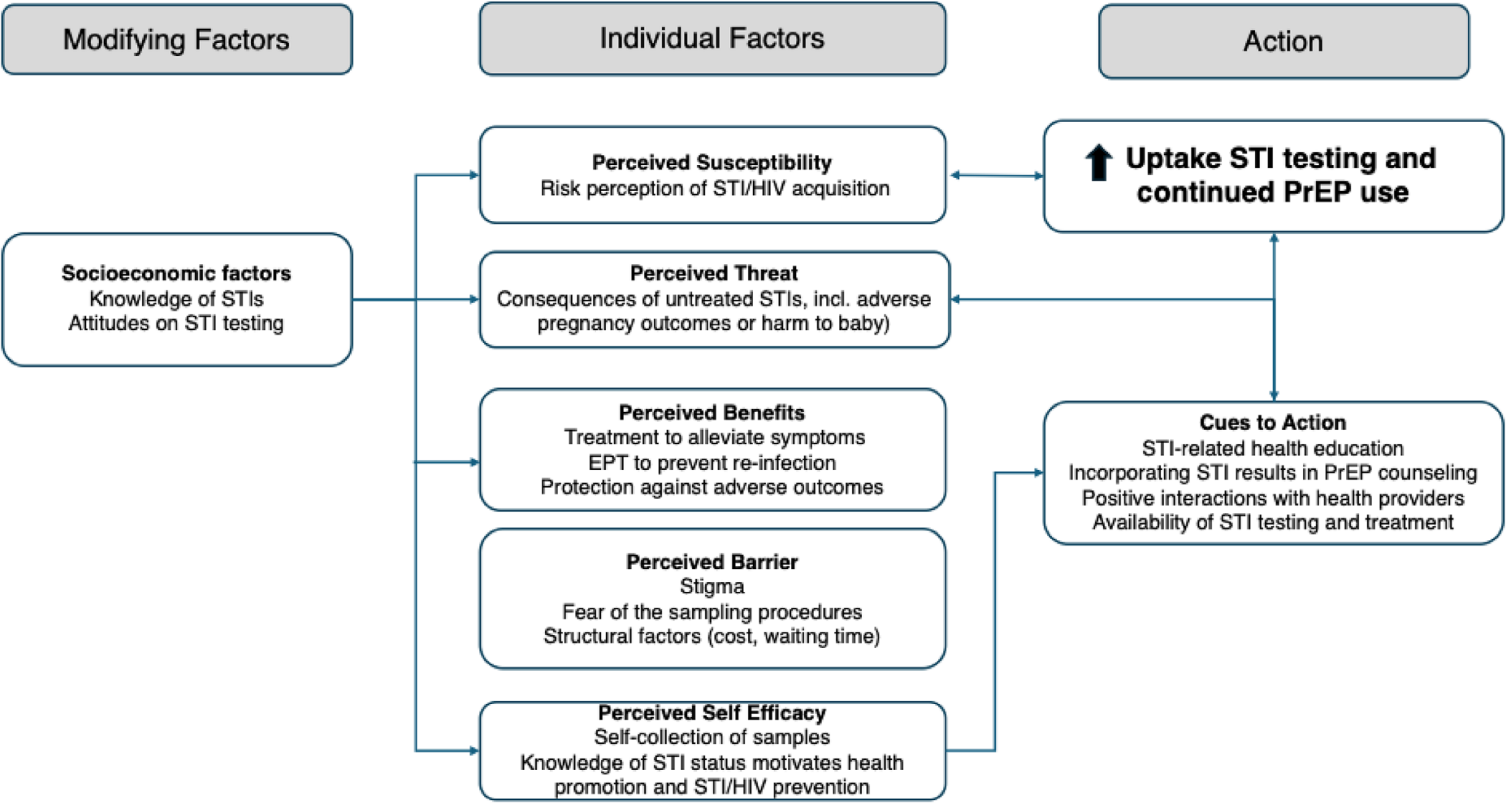
Women's experiences and motivations for STI testing uptake and continued PrEP use based on the Health Belief Model, adapted from Rosenstock et al. [24]. EPT, expedited partner therapy; PrEP, pre‐exposure prophylaxis; STI, sexually transmitted infection.

In total, 39 women who initiated PrEP in pregnancy, and tested for CT and NG, participated in IDIs. Six participants tested positive for an STI (four for CT, one for NG, one for both); all received directly observed treatment and accepted EPT for their sexual partners. The median age was 26 years (interquartile range [IQR]: 21–29), 28 (72%) were married, 30 (77%) had >8 years of completed education and 6 (15%) were employed. At 9 months postpartum, 82% persisted with PrEP use (e 1). Related to the Health Belief Model, we identified several key themes (Figure 1), including positive experiences with STI testing and factors that facilitated testing (*perceived benefits and health motivation domains*), contextualizing HIV/STI risk through STI testing (*perceived vulnerabilities*) and how STI testing influenced PrEP use (*cues to action*). We additionally summarized experiences with EPT due to the unique circumstances of these participants.

**Table 1:** Characteristics of participants who initiated PrEP during pregnancy, tested for CT/NG and participated in in‐depth interviews (*n* = 39)

Variables	*n* (%) or median (IQR)
**Demographic characteristics**	
Age (years)	26 (21−29)
Years of completed education	12 (10−13)
Employed (vs. unemployed)	6 (15%)
**Relationship characteristics**	
Currently in a relationship	38 (97%)
Marital status	
Married/co‐habiting	28 (72%)
Single/non‐cohabiting	11 (28%)
Marriage type polygamous (vs. monogamous)	4 (14%)
**Partner characteristics** (*n* = 32)	
Partner age difference ≥ 5 years (vs. <5 years)	13 (41%)
Partner HIV status	
HIV negative	5 (14%)
Unknown status	34 (86%)
**Clinical characteristics**	
Positive CT and/or NG test	6 (15%)
Partner accepted EPT^a^ (*n* = 6)	6 (100%)
PrEP status at 9 months postpartum	
Continued	32 (82%)
Discontinued	7 (18%)
**Risk assessment characteristics**	
Empiric HIV risk score^b^	9 (8−9)
Number of lifetime sexual partners	3 (2−3)
Partner has other sexual partners^c^ (*n* = 38)	11 (29%)

Abbreviations: CT, *Chlamydia trachomatis*; EPT, expedited partner therapy; IQR, interquartile range; NG, *Neisseria gonorrhoeae*; PrEP, pre‐exposure prophylaxis.

^a^ Among those testing positive and accepting EPT (*n* = 6).

^b^ Empiric HIV risk assessment to predict HIV incidence in pregnancy and postpartum (HIV risk score ≥ 6) (translating to HIV incidence 7.3 per 100 person‐years).

^c^ Among those in a relationship (*n* = 38).

### 3.2 Perceived vulnerabilities to STI and benefits of STI testing

Some women perceived themselves to be at high risk for contracting STIs due to polygamous marriages, suspecting their partners had other sexual partners, or women themselves having multiple sexual partners. Perceiving STI risk related to these factors motivated some women to test for STIs as reported by one woman below:

“So, I had three different sexual partners, all of them knew that the pregnancy was theirs [giggles]… I didn't know their sexual behaviors too. So, I had to test to find out whether I was infected [with STIs] or not.”—**Age 28, CT/NG negative, continued PrEP**.

“There are many diseases around, …with how our men nowadays sleep around, you can't know their health status. I usually just stay back at home, due to the nature of my work, but I don't trust my partner's movements [suscepts infidelity]. …he usually returns home very late and doesn't say where he is. So, l decided to test.”—**Age 28, CT/NG negative, discontinued PrEP**.

Women demonstrated varied STI knowledge, with most women being familiar with examples such as chlamydia, gonorrhoea, chancroid, and syphilis and their related symptoms, such as vaginal itching and abnormal vaginal discharge. Some women accepted STI treatment upon experiencing symptoms related to CT/NG, such as vaginal itching and abnormal discharge. Women acknowledged that untreated STIs could potentially lead to complications, including difficulty in conception, stillbirths/miscarriages and preterm births, although the depth of awareness was varied. Others suggested that beyond pregnancy, a baby may be born with a mental disorder or disability, or the mother may develop complications if STIs are left untreated.

“… Gonorrhea, syphilis, are the ones that I know…If a pregnant woman gets it, that would be very bad… It can cause miscarriage, you can also give birth to [an] unhealthy baby. That disease [STI] is bad, it can be good luck if the baby comes out alive since it can cause stillbirth. The fetus might not have good health in the womb”—**Age 28, CT/NG negative, discontinued PrEP**.

The availability of CT and NG testing within the antenatal clinics, coupled with health education from providers, motivated women to test. While most women were aware of syphilis testing for pregnant women, they were unaware that testing for other STIs was possible. Discussions held with the providers sensitizing women on the importance of testing, implications for delayed testing and the availability of treatment if a test result was positive were motivators for women to test.

“I knew if I tested, then I would be free because I had never tested for [STIs], so when I came here and found that they were testing, I found it to be good. Now you live knowing your status. You might be infected and you don't know, they [STIs] can eat someone from inside until it reaches a point where they cannot even give birth… So that was the reason why I decided to take the test.”—**Age 33, CT/NG negative, continued PrEP**.

Having a positive interaction with providers was highlighted as a motivator for STI testing, as providers within the maternal and child health clinics were friendly, welcoming and non‐judgemental. One participant had symptoms but feared discussing it at the general outpatient clinic; however, healthcare providers at the antenatal clinic were warm and approachable, prompting her to disclose her symptoms and agree to test.

“In outpatient, I was afraid because I didn't have someone I can tell my problems. When I joined the study, they [providers] were friendly and …. I told them I had some itching and some yellowish discharge, and they asked if I would agree to be tested. Which I accepted, so that is how I accepted to be screened.”—**Age 20, CT/NG positive, continued PrEP**.

Some women feared that collecting the sample for STI testing would hurt their baby; however, following reassurances from providers that the process was simple and painless, receipt of a detailed description of sample collection procedures and options for provider‐assisted sample collection, women became more at ease. Women also recognized the importance of early detection and treatment of STIs to prevent poor outcomes.

“Before she gave me the sample collecting tube, [the nurse] took me through STIs, how they manifest and what would happen in case you could be having one, and how I would be helped in case I have one…this made me become courageous while undergoing these procedures. …because in case I tested positive, then I could get immediate help… that is why I decided to take all these initiatives. If I tested positive, then I can get help early enough.”—**Age 27, CT/NG negative, continued PrEP**.

### 3.3 Cues to action: STI testing and PrEP use

Most women stated that testing for CT/NG did not negatively influence their PrEP adherence since they still considered themselves to be at risk for HIV, regardless of their CT/NG test result. Some women expressed that negative STI results encouraged health‐promoting behaviours, including sustained PrEP use, even when STI results were negative, as testing made women feel proactively involved in preventing HIV/STI complications for themselves and their infants.

“It motivated me because when I realized that I am not infected with STI, I was trying hard not to get HIV, so from that point, I made a decision to continue with the PrEP; I did not leave [stop taking PrEP], even after the results [STI] came out negative.”—**Age 21, CT/NG negative, continued PrEP**.

Women who tested positive for CT/NG expressed a heightened perception of risk for other STIs, including HIV, and the test results motivated them to continue PrEP.

“Yes, I felt I was at risk. Because now that I have this STI, chances are high that I also get infected with other STIs. For example, if I have sex with another man and I don't know their status. I can contract it again. This showed that I could be at risk of getting HIV and other STIs, and that is why I made a decision to change my behavior….”—**Age 20, CT/NG positive, continued PrEP**

One participant in a polygamous marriage still considered herself at risk despite testing negative for CT/NG due to the added risk of infection from the other partners, thus motivated to continue PrEP.

“…I would have wished that my spouse and my co‐wife all get tested [for STI]… I can be [negative], but one of them [sexual partner or co‐wife] has it [STI]… without knowing the other people's status would make my chances of contracting it [STI] higher. Because… I was [tested] negative [for STI], this really encouraged me to continue taking PrEP… to be [HIV‐negative] and give birth to an infection‐free baby”—**Age 27, CT/NG negative, continued PrEP**.

### 3.4 Experiences with EPT

Women expressed disappointment, feelings of shame and perceived stigma following a positive STI result. Some feared their partner's responses to their positive test, while others felt they had been betrayed by their partners. All women testing positive were offered and accepted directly observed therapy and EPT; however, most were anxious regarding delivering EPT to partners.

“I felt bad because I didn't expect that I shall have an STI. I didn't understand where it came from, was it an infection from my husband or… It worried me. He was also not comfore and asked me what was the cause. The challenge that I had was convincing him to take the drugs…”—Age 20, CT/NG positive, continued PrEP.

“I was scared because I was asking myself if I go with these drugs, how will I tell him for him to also agree and take, so I feared. He just asked me, ‘how did you contract it [STI]?’ and I told him I am the one who is supposed to ask you.”—Age 22, CT/NG positive, discontinued PrEP.

While women recognized the importance of treating their sexual partners before resuming sex to prevent re‐infection, some encountered challenges in relaying the test results and dispensing EPT, often employing a variety of options such as persuasive language, deception or confrontational language to induce uptake and completion of EPT.

“… I told him that since I am pregnant and we can still be intimate, I don't want to affect my baby in case he or I have other partners. I took the first drug in his presence, and he also did so.”—**Age 20, CT/NG positive, continued PrEP**.

### 3.5 Recommendations for improving STI testing user experience and EPT

Some women were uncomfore with sample collection, citing a lack of prior experience and fear of inserting the swab into the vagina. However, following support from a provider, they successfully collected the sample.

“I was not comfore because it involved insertion of items in the vagina. I had no prior experience with it and feared a little bit.”—**Age 27, CT/NG positive, continued PrEP**.

Satisfaction was high among women who used private rooms for sample collection, while those using facility toilets had mixed reactions, some citing concerns about privacy, cleanliness and long queues. Several women suggested provider‐assisted services, including guided sample collection instructions or providers collecting samples for those fearful or unable to collect samples, while others recommended learning self‐sample collection to reduce healthcare providers’ workload.

“I can say it's better you collect the sample by yourself so that you can learn, like next time when you go back again, the nurse will not have a lot of work, so it's you who will do for yourself.” **Age 22, CT/NG positive, discontinued PrEP**.

Most women agreed that routine STI testing should be “opt‐out,” similar to routinized HIV testing in antenatal care. Testing all women was encouraged since some women might be fearful to request a test, and others might lack symptoms. While some women were comfore with testing at enrolment and after delivery, some suggested more frequent testing would be beneficial to ensure treatment before complications ensue.

“They should perform [STI testing] for all women who are pregnant. Even after delivery, testing should… continue. They should make [testing] mandatory so that everyone knows their status. They should copy the way they always do for HIV testing. When you come to the facility, they direct you to the screening room where they collect our samples, if they can copy that.”—**Age 31, CT/NG positive, continued PrEP**.

In contrast, a few participants suggested that women should be asked before testing and that the test should only be given to those who accept. Some women were not aware of the STI testing services until they were offered the test as part of the study activities and recommended health education to all women seeking routine services within the clinic. Some women suggested the provision of free treatment services as a motivation for routine screening.

“…one might turn positive for STI but don't have money to treat it. They [hospitals] should offer [free] treatment to motivate us to get tested. During physical clinic visits, they [providers] should create time to offer health education in a group, not only individually.”—Age 32, CT/NG negative, discontinued PrEP.

“…when sensitizing people on STI, … consider that some people are shy and will not accept testing. Some don't know how to collect the sample but wish to be tested. But if you sensitize them well, they will accept even if it is the nurse collecting samples.”—Age 23, CT/NG negative, continued PrEP.

## 4 DISCUSSION

This qualitative study evaluated women's experiences with CT/NG testing within antenatal clinics delivering PrEP and the impact of CT and NG testing on PrEP use. Factors that motivated testing included perceived vulnerabilities to STIs and benefits of testing, similar to studies on screening for other conditions (e.g. diabetes, hypertension and breast cancer) [32, 33, 34]. These findings underscore the importance of educating pregnant women on the risks of STIs and the benefits of screening to promote STI testing uptake, highlighting an opportunity for antenatal education. Positive interactions with providers and the availability of STI treatment also motivated STI testing. STI testing refined risk assessment and encouraged PrEP use, even when STI results were negative. Our results complement prior pilot evaluations which found high acceptability of antenatal STI testing assessed quantitatively by adding qualitative perspectives from end‐users and identifying opportunities for strengthening co‐delivery of STI and HIV prevention services for pregnant women [15, 25, 35].

In our study, STI testing encouraged health‐promoting behaviours, including sustained PrEP use, even when STI results were negative, as testing made women feel proactively involved in preventing HIV/STI complications for themselves and their infants. Moreover, women in polygamous marriages and those who had multiple partners (or partners with multiple partners) remained motivated to continue PrEP as a means of protection, even after negative STI results. For those who tested positive, STI testing confirmed self‐perceived risk and further motivated PrEP use. Relationship dynamics and power imbalances may lead to an inability to negotiate safer sex practices, thus influencing perceived risk and utilization of HIV/STI prevention strategies that are within one's reach and control [36, 37, 38]. Serial STI testing during periods of PrEP use could allow women to re‐evaluate and/or recognize their risks to proactively make informed decisions about STI/HIV prevention [39].

Studies among pregnant women in South Africa and Kenya suggest STIs are common during pregnancy, and STI diagnosis motivates PrEP initiation [25, 40, 41]. For instance, recent findings among pregnant women not living with HIV in South Africa found that having an STI diagnosis and/or symptoms was associated with higher proportions initiating oral PrEP compared to lack of an STI diagnosis or symptoms [40, 42]. These findings indicate that STI testing may influence PrEP use in pregnancy. Yet, there is a paucity of data on serial STI testing in pregnancy through the postpartum period to promote sustained PrEP use. Future studies are needed to examine if serial STI testing promotes consistent PrEP use through pregnancy and postpartum.

Positive interactions with healthcare providers, receiving comprehensive STI education and accessible STI services, including immediate treatment and EPT, all promoted STI testing in our study. Providers have a pivotal role in the uptake of new STI services or strategies since they are the main contact with patients [35, 43, 44]. Social norms reinforcing stigma, discrimination or perceived hostility from providers associated with being diagnosed with STIs may lead some women to avoid testing services, thus limiting uptake [16, 45]. Creating safe spaces within the healthcare setting can empower patients to prioritize their health and access STI testing and other reproductive health interventions [35, 43, 44]. Prior studies demonstrate the effectiveness of incorporating shared decision‐making in disease management [46].

In our study, women knew the risks associated with STIs in pregnancy, but few were aware of strategies to prevent and manage STIs, including diagnostic testing. Education to increase awareness of STI complications and management in pregnancy may facilitate informed decision‐making on using STI services among antenatal care patients. Increasing the availability of STI testing within antenatal care, coupled with immediate treatment and offer of EPT, would also facilitate reducing the STI burden in this population. To date, cost has limited the availability of empiric STI testing services beyond HIV and syphilis. Scaling up low‐cost point‐of‐care diagnostics for other STIs is urgently needed in antenatal care.

Routinizing STI testing as “opt‐out” would increase coverage and potentially address the stigma attached to the current standalone STI services delivery approach [16]. “Opt‐out” testing is a CDC recommendation for HIV testing that presumes an individual has consented unless they explicitly decline and is often used in regions of high HIV burden [47]. Studies in Kenya consistently show universal “opt‐out” testing is accepe and increases HIV testing [48, 49]. The WHO recommends incorporating STI services alongside other routine health services to reduce cost, increase access and uptake [50]. Integrating point‐of‐care STI testing into antenatal care settings can facilitate same‐day treatment of both women and their sexual partners, thus disrupting the cycle of reinfections [14, 19, 51].

Our study had a few limitations. The parent study enrolled pregnant women with high HIV risk scores who initiated PrEP within routine antenatal care clinics who may experience STI testing differently compared to general populations of pregnant women or those who decline PrEP. Our sample did not include women who initiated PrEP in settings outside of antenatal clinics, though >80% of women in Kenya attend public antenatal clinics [52]. A dedicated team of study nurses and laboratory technicians provided health education and guided the sample collection process. We did not assess baseline STI knowledge before initiating STI testing; therefore, cannot distinguish STI knowledge before and after study participation. The level of knowledge among our study participants may not represent the general population's knowledge on STIs. The quality of services provided by the study staff and the provider‐patient relationship may be different from that of routine staff in public health clinics. Women were purposively selected based on co‐offer of STI testing. Data suggest that co‐offering STI testing with PrEP may promote PrEP use [1, 2], which may account for the higher rate of PrEP persistence (82%) in the subgroup of women offered STI testing in this qualitative analysis compared to other studies of postpartum PrEP users.

## 5 CONCLUSIONS

Our qualitative evaluation found that STI testing is accepe when co‐delivered with PrEP for pregnant women. Health Belief Model constructs encouraged uptake of both STI testing and continued PrEP use. Factors that motivated STI testing among women who initiated PrEP in pregnancy included positive interactions with providers and receipt of STI education (*cues to action and perceived self‐efficacy*) that reinforced the benefits of testing and availability of STI treatment and EPT (*perceived threats and benefits*). Additionally, STI testing encouraged PrEP use, even when STI results were negative. Integrating STI testing and PrEP delivery services within antenatal care represents a potential “high yield” opportunity to engage women in discussions targeting HIV and other STI prevention strategies in pregnancy.

## COMPETING INTERESTS

No competing interests to declare.

## AUTHORS’ CONTRIBUTIONS

GJ‐S, JK, JP and JNM conceptualized and designed the study. TS, EA and JNM developed data collection tools, while KN, JP, KMB‐S and MM reviewed and provided feedback on the data collection tools. TS, FA, EA and JNM participated in training and oversaw the data collection processes. EA, TC, HA, SO and JNM participated in data analysis. ARW, CM, GJS, AM, JP and JNM participated in the drafting and revision of the paper. All authors critically reviewed and approved the final manuscript.

## FUNDING

This study was funded by the National Institute of Nursing Research (R01NR019220) with support from the University of Washington Center for AIDS Research (P30 AI027757). JP was additionally supported by the Eunice Kennedy Shriver National Institute of Child Health & Human Development (NICHD, R01HD108041, R01HD100201, R01HD113455 and R01HD117702). The mWACh‐PrEP Study Team was supported by the University of Washington's Global Center for Integrated Health of Women, Adolescents, and Children (Global WACh). The funders had no role in study design, data collection and analysis, decision to publish or manuscript preparation.

## Data Availability

Data that support the findings of this study are available on request from the corresponding author.
